# Genome-Wide Identification of the *Hsp20* Family Responding to Heat Stress in Sunflower (*Helianthus annuus* L.)

**DOI:** 10.3390/ijms27135799

**Published:** 2026-06-26

**Authors:** Yushan Liu, Shurui Dong, Qian Zhang, Wenning Liu, Xiaolei Wu, Cheng Lu, Leyang Wu, Ye Sun, Jing Liu, Maohong Cai, Tao Chen

**Affiliations:** School of Life and Environmental Science, Hangzhou Normal University, Hangzhou 311121, China; 2023111010006@stu.hznu.edu.cn (Y.L.); 2024111010036@stu.hznu.edu.cn (S.D.); 2024112010044@stu.hznu.edu.cn (Q.Z.); 2024111010050@stu.hznu.edu.cn (W.L.); 2025112010056@stu.hznu.edu.cn (X.W.); 2025112010014@stu.hznu.edu.cn (C.L.); 2025112010023@stu.hznu.edu.cn (L.W.); 2025111010014@stu.hznu.edu.cn (Y.S.); 2025111010025@stu.hznu.edu.cn (J.L.)

**Keywords:** sunflower, *Hsp20* gene family, heat stress, expression analysis

## Abstract

Small heat shock proteins (Hsp20s) function as essential molecular chaperones in plant stress responses, yet their genome-wide characterization in sunflower (*Helianthus annuus* L.) remains lacking and their functional role in heat response is also unknown. In this study, 65 *HaHsp20* genes were identified in sunflower through a comprehensive genome-wide analysis based on the conserved ACD (α-crystallin) domain. The expansion of this family was primarily driven by whole-genome duplication (WGD) or segmental duplication events, with the CI subfamily (20 members) representing the most significantly expanded lineage-specific clade. While all HaHsp20 proteins harbor the conserved α-crystallin domain (ACD), they exhibit diverse molecular weights (11.31–53.35 kDa), isoelectric points (4.71–9.75), and subcellular localization patterns. Promoter cis-regulatory element analysis revealed a predominance of ABA and MeJA-responsive elements but only two canonical heat shock elements. Transcriptome and RT-qPCR analyses revealed that most *HaHsp20* genes are responsive to heat stress, with seven *HaHsp20* genes exhibiting extremely upregulated expression (more than 1000-fold) after 10 h of 45 °C treatment. Among these, *HaHsp21.59* and *HaHsp25.91* showed an increase of over 4000-fold in expression. These findings provide a comprehensive foundation for understanding the evolutionary history and expression dynamics of the *HaHsp20* family in sunflower, and highlight *HaHsp21.59* and *HaHsp25.91* as promising candidate genes for future functional validation of their potential roles in heat stress tolerance.

## 1. Introduction

Heat stress severely restricts plant growth and development [1]. At the individual level, high temperature inhibits seed germination. For example, the germination rate of sunflower seeds is significantly reduced at 40 °C [2]. It causes leaf wilting and scorching in seedlings, excessive hypocotyl elongation, and impaired root growth, thereby reducing water and nutrient uptake and decreasing biomass accumulation [3,4]. During the reproductive stage, high temperature induces pollen sterility and impairs fertilization, directly reducing crop yield [5,6,7]. At the grain-filling stage, high temperature inhibits photosynthesis and grain filling, affecting both yield and quality [8,9]. At the cellular physiological level, high temperature disrupts chloroplast structure, damages PSII function, impedes electron transport, and exacerbates photoinhibition; heat-induced stomatal closure limits CO_2_ supply, while increased respiration leads to carbon loss [10,11,12]. Simultaneously, high temperature triggers a burst of reactive oxygen species (ROS), which attack membrane lipids, proteins and nucleic acids, causing oxidative damage, and alter membrane fluidity, resulting in malondialdehyde (MDA) accumulation, further interfering with key physiological processes such as photosynthesis [13,14,15,16]. At the molecular level, the membrane-associated NAC transcription factor *OsNTL3* is activated and translocated to the nucleus under heat stress, where it upregulates the expression of genes involved in ER protein folding and ROS scavenging, thereby enhancing rice thermotolerance [17]. The WRKY transcription factor SlWRKY3 positively regulates tomato thermotolerance by directly binding to the promoter of the ROS-scavenging gene cluster *SlGRXS1*, reducing ROS accumulation [18]. The MYB transcription factor MYB44 interacts with ABI5, which weakens MYB44 binding to the *PPH* and *PAO* promoters and promotes ubiquitin-dependent degradation of MYB44, accelerating heat-induced chlorophyll degradation [19]. Beyond NAC, MYB, and WRKY, heat shock transcription factors (HSFs) also play a key role in activating heat stress responses. HSFs trigger a transcriptional cascade that upregulates stress-responsive genes, especially heat shock proteins (Hsps) [20].

Heat shock proteins (Hsps), as molecular chaperones, play a central role in maintaining cellular protein homeostasis. When plants are subjected to stress, Hsps are rapidly upregulated to protect other proteins from stress-induced damage, thereby preserving intracellular protein homeostasis [21]. Hsps are stress proteins synthesised at specific developmental stages during plant evolution to adapt to different living environments and they represent a special self-defence mechanism in plants [22]. According to molecular weight, Hsps are generally divided into five subfamilies: Hsp100, Hsp90, Hsp70, Hsp60 and Hsp20. Among them, Hsp20 proteins (also known as small heat shock proteins, sHsps) are considered the most abundant and evolutionarily most complex family and are of great significance in high-temperature stress adaptation and crop thermotolerance improvement [23]. Compared with other Hsp subfamilies, members of the Hsp20 subfamily generally have smaller molecular weights, mostly ranging from 12 to 42 kDa. A comprehensive understanding of the structure, function and regulation of the *Hsp20* gene family will help elucidate the thermotolerance mechanism of plants.

The most central function of sHsps is to act as ATP-independent molecular chaperones. From the structural perspective, sHsps are characterised by a highly conserved α-crystallin domain (ACD) of approximately 90 amino acid residues in the C-terminal region, which directly participates in substrate recognition and binding and plays a key role. The ACD is flanked by a variable N-terminal domain and a short C-terminal extension. The N-terminal domain is thought to be involved in oligomer assembly and substrate-specific binding, while the C-terminal extension regulates oligomer stability and subunit exchange, conferring functional diversity to different members [24,25]. The number and diversity of sHsps in plants are far greater than in animals and fungi, and this expansion is closely related to the sessile lifestyle of plants and their inability to escape environmental stresses on land. Based on subcellular localisation, plant sHsps can be divided into multiple subclasses [25,26]. Members of the *Hsp20* gene family have been studied in many plants. Transgenic *Arabidopsis thaliana* lines overexpressing *DcHSP20-12* exhibited significantly higher germination rates, fresh weight, and root length, but lower malondialdehyde (MDA) content under heat stress, indicating enhanced thermotolerance [27]. Knockout of *ZmHSP20-5* in maize impairs its root structure, resulting in reduced drought resistance [28]. Silencing of *CaHsp25.9* in the heat-tolerant pepper line R9 led to increased accumulation of MDA, hydrogen peroxide, and superoxide anions under heat, salt, and drought stress. This suggests that *CaHsp25.9* enhances tolerance to these stresses by reducing reactive oxygen species accumulation, boosting antioxidant enzyme activities, and regulating stress-related gene expression [29]. These findings demonstrate that the *Hsp20* family has undergone remarkable expansion and subfunctionalization in plants, with functions extending beyond classical heat tolerance to responses to drought, salt, oxidative stress, pathogen infection, and many other adverse conditions [30,31,32].

Sunflower (*Helianthus annuus* L.) is one of the world’s most important oilseed crops. Its entire growth period mostly coincides with the hottest time of the year. As global warming continues, the impact of heat stress on sunflower growth is becoming increasingly significant, often leading to reduced seed set, decreased oil content, and serious limitations on yield and quality [33]. In 2017, the release of a high-quality sunflower reference genome made it possible to systematically identify and characterise stress-related gene families at the genome-wide level [34]. In sunflower, several heat-responsive factors have been identified, including members of the *HSF* and *Hsp* families as well as the transcription factor *HaHB4*, all of which are involved in heat shock signal transduction and protein protection. For example, Ceylan et al. analyzed the expression patterns of the sunflower *HSF* and *Hsp* families under drought and salt stresses; however, their study did not specifically investigate the heat-response patterns of the *Hsp20* family [35]. In addition, heterologous expression of *HaHB4* in wheat was shown to significantly enhance heat tolerance at the pre-anthesis stage [36]. Although the *Hsp20* family has been relatively well studied in other crops such as rice [37] and soybean [38], its expression profiles and functional roles under heat stress in sunflower remain largely unexplored. Therefore, in-depth mining of *HaHsp20* gene family members, determination of their gene structures, chromosomal distribution patterns, and promoter cis-regulatory element (CRE) features, and systematic analysis of their expression patterns under high-temperature and other stress conditions will not only help reveal the sunflower-specific molecular mechanism of heat-responsive expression but also provide direct theoretical support and genetic resources for stress-tolerant molecular breeding.

In this study, we systematically identified and characterized the *HaHsp20* gene family in sunflower, including analyses of physicochemical properties, conserved domains, gene structures, phylogeny, synteny, promoter cis-regulatory elements (CREs), and tissue-specific expression profiles, along with an examination of their transcriptional responses to high-temperature stress. Through combined transcriptomic and RT-qPCR approaches, we reveal that several *HaHsp20* genes exhibit exceptionally strong heat responsiveness, with seven genes showing more than 1000-fold induction after 10 h at 45 °C, among which *HaHsp21.59* and *HaHsp25.91* displayed the most dramatic upregulation, exceeding 4000-fold. These findings establish a comprehensive foundation for understanding the evolutionary dynamics and heat-responsive expression of the *HaHsp20* family in sunflower, and provide a prioritized set of candidate genes for future functional validation and potential application in breeding for heat stress tolerance.

## 2. Results

### 2.1. Identification and Analysis of HaHsp20 Family

*HaHsp20* genes play important roles in response to heat; to identify the *HaHsp20* genes in sunflower, the conserved Hsp20 domain (PF00011) was used for analysis and a total of 65 *HaHsp20* genes were identified in sunflower. These genes were subjected to physicochemical property analysis and named according to their molecular weights (Table 1). The results showed that the amino acid lengths of HaHsp20 proteins ranged from 100 aa (HaHsp11.31) to 462 aa (HaHsp53.35), with corresponding molecular weights ranging from 11.31 kDa (HaHsp11.31) to 53.35 kDa (HaHsp53.35). The predicted isoelectric points (pI) of the HaHsp20 proteins ranged from 4.71 (HaHsp20.03) to 9.75 (HaHsp36.14), among which 25 proteins were basic (pI > 7). The GRAVY (grand average of hydropathicity) values ranged from −1.097 (HaHsp26.49) to −0.068 (HaHsp20.03), all of which were negative, indicating that all HaHsp20 proteins were strongly hydrophilic. Subcellular localization prediction revealed that 37 HaHsp20 proteins are localized in the cytoplasm, 3 in the nucleus, 18 in the chloroplasts, 3 in the mitochondria, 1 in the Golgi apparatus, and 3 in the peroxisomes. These results suggest that Hsp20 proteins may function in various cellular environments.

### 2.2. Phylogenetic Analysis of HaHsp20 Proteins

**Figure 1:**
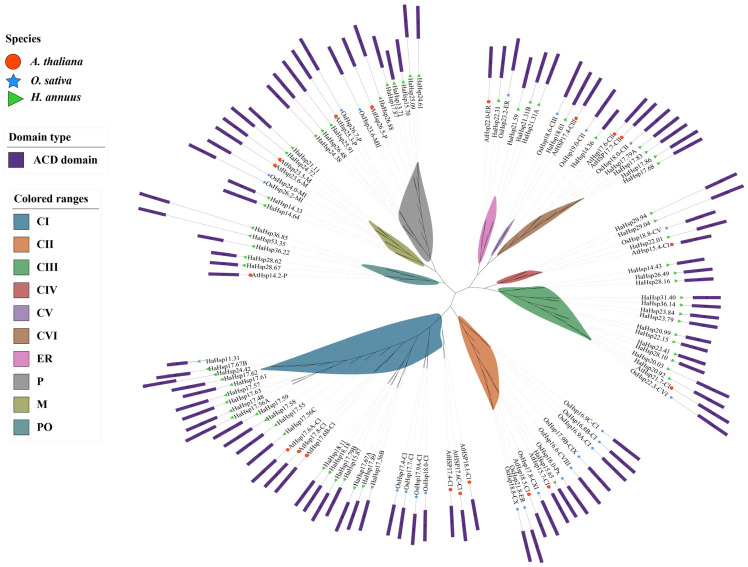
Phylogenetic tree of Hsp20 proteins from *Arabidopsis thaliana*, *Oryza sativa*, and *Helianthus annuus*. Phylogenetic analysis of the Hsp20 protein family in sunflower and other plant species. At, *Arabidopsis thaliana*; Os, *Oryza sativa*; Ha, *Helianthus annuus*. Different colors represent different subfamilies. CI-CVI indicate proteins localized in the cytoplasm; P and PO indicate proteins localized in peroxisomes; M indicates proteins localized in mitochondria; ER indicates proteins localized in the endoplasmic reticulum.

To investigate the phylogenetic relationships and structural diversity of the *Hsp20* gene family, a maximum-likelihood phylogenetic analysis was performed using the Hsp20 protein sequences from *Arabidopsis* (19), rice (23) and sunflower (65) (Figure 1). Based on subcellular localization analysis, the HaHsp20 proteins were classified into 10 subfamilies: CI-CVI (cytoplasmic localization), ER (endoplasmic reticulum localization), P and PO (peroxisomal localization), and M (mitochondrial localization). Among them, CI contained the largest number of HaHsp20 proteins (*n* = 20), whereas CII and CV contained the smallest number (*n* = 1 each). In these 10 groups, the PO consisted exclusively of HaHsp20 proteins from dicot species, whereas the other groups included members from both dicot and monocot lineages. The clustering pattern of HaHsp20 proteins was largely consistent with their predicted subcellular localization from WoLF PSORT, with the majority localized in the cytoplasm, suggesting that they primarily function there.

### 2.3. Gene Structure and Conserved Motif Analysis of HaHsp20

**Figure 2:**
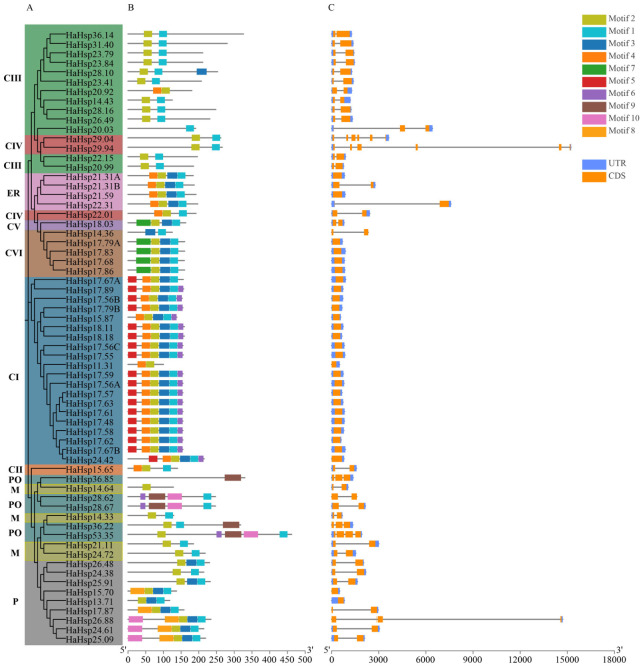
Phylogenetic relationships, conserved protein motifs, and gene structure composition of *HaHSP20* genes. (**A**) Maximum likelihood (ML) phylogenetic tree of HaHsp20 proteins. (**B**) Distribution of conserved motifs in HaHsp20 proteins identified by MEME analysis. The ten identified motifs (Motif 1–Motif 10) are represented by different colored boxes, and their relative positions are indicated. The scale bar at the bottom indicates protein length (amino acids). (**C**) The gene structure of *HaHsp20* genes. Orange boxes represent coding sequences (CDS), and blue lines represent introns. The scale bar at the bottom indicates gene length (base pairs).

To further dissect the sequence characteristics and structural diversity of the sunflower *HaHsp20* gene family, conserved motifs were predicted in the HaHsp20 protein sequences, and the exon-intron structures of the corresponding genes were analyzed using genomic information. Ten significantly conserved motifs (Motif 1 to Motif 10) were identified in the 65 HaHsp20 proteins using the MEME tool (Figure 2A,B). Motif 1 and Motif 2 were universally present in all subclades, forming the core backbone of the HaHsp20 proteins. Notably, the ER group contained Motif 1, Motif 2, Motif 3, and Motif 4, suggesting that these motifs may be closely associated with the unique functions of this subclade. Motif 10 was detected in both the P and PO groups, both of which are predicted to localize to peroxisomes, implying a potential role in regulating basic cellular physiological activities. In addition, several motifs exhibited group-specific distribution patterns. For example, Motif 9 was detected only in the PO group, Motif 5 exclusively in the CI group, and Motif 8 only in the P group.

Gene structure analysis revealed that among the 65 *HaHsp20* genes, *Hsp29.94* exhibited the largest genomic span. Moreover, *HaHsp29.04* and *HaHsp29.94* displayed the most complex structures, each containing six exons and five introns (Figure 2C). In addition, among the 65 *HaHsp20* genes, 28 were intron-less (single-exon) genes, 30 contained two exons, 3 contained three exons, and 2 contained four exons.

### 2.4. Localization of HaHsp20 Genes on Chromosomes and Syntenic Analysis

**Figure 3:**
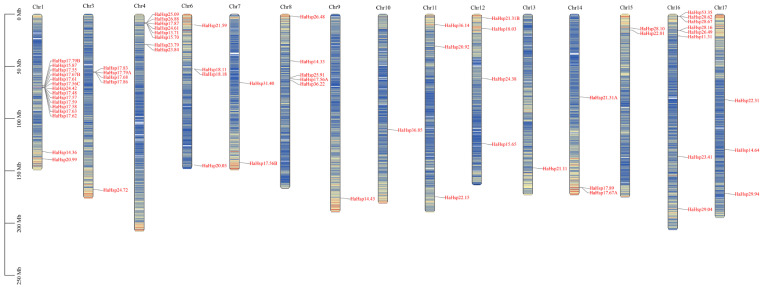
Genomic localization of the *HaHsp20* gene family in sunflower. Positions of the 65 *HaHsp20* genes on sunflower chromosomes. Only chromosomes containing *HaHsp20* genes are shown. Gene positions are indicated in megabases (Mb), and gene IDs are highlighted in red.

Chromosomal distribution analysis revealed that the 65 genes were unevenly distributed across 17 chromosomes (Figure 3). Chromosome 1 exhibited the highest gene density, harboring 15 *HaHsp20* genes, followed by chromosomes 4 and 16, each containing eight genes. Chromosomes 9, 10, and 13 each contained only one *HaHsp20* gene, whereas chromosomes 2 and 5 had no *HaHsp20* genes. The remaining chromosomes carried between two and five genes.

**Figure 4:**
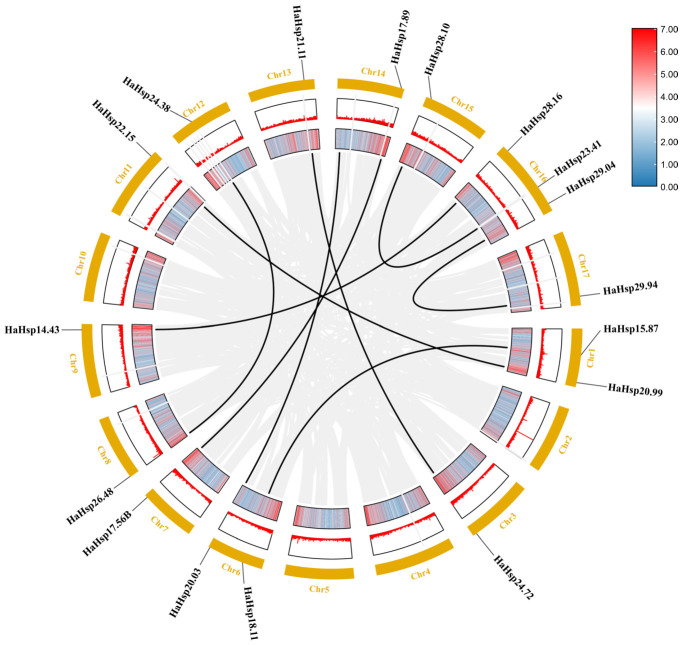
Synteny analysis of *HaHsp20* genes in sunflower. Highlighted black lines represent homologous relationships between pairs of *HaHsp20* genes. The gene identifiers of each syntenic pair are annotated next to their respective chromosomal positions.

To further understand the expansion and evolution of this gene family, intraspecific synteny analysis of the *HaHsp20* family genes was performed in sunflower. The results showed that syntenic relationships were unevenly distributed across 13 chromosomes, with a total of nine syntenic gene pairs identified (Figure 4). Tandem duplications were defined as two or more homologous genes located within 200 kb on the same chromosome with no more than one unrelated gene between them, whereas segmental duplications were identified based on homologous blocks detected by MCScanX under default parameters [39]. All duplicated genes identified were derived from whole-genome duplication (WGD) or segmental duplication events (Figure 4). To evaluate the selective pressures acting on duplicated *HaHsp20* genes, we calculated the Ka/Ks ratios for all paralogous pairs identified (Table 2). All Ka/Ks values were substantially below 1, ranging from 0.121 to 0.403, indicating that these gene pairs have evolved under strong purifying selection and are subject to strict functional constraints. The Ks values varied widely, ranging from 0.367 to 1.337, suggesting that the duplication events occurred at different evolutionary time points.

### 2.5. Tissue Expression and Promoter Analysis of HaHsp20 Genes

**Figure 5:**
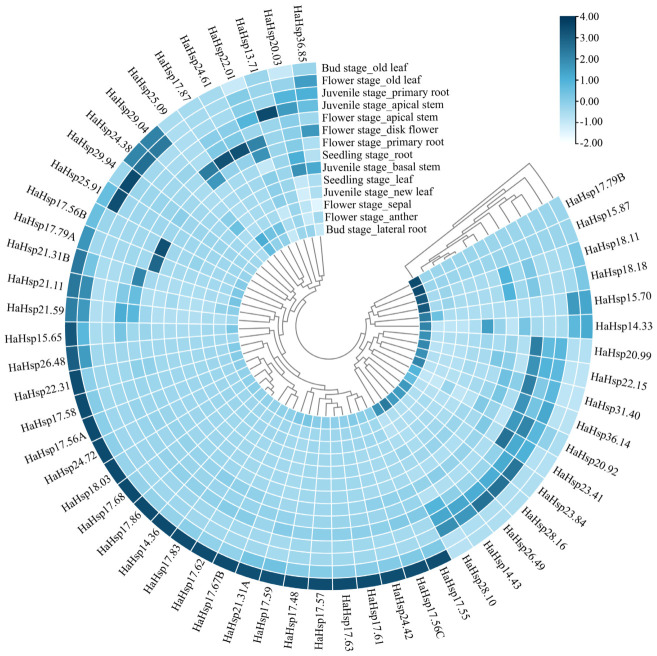
Tissue-specific expression patterns of the *HaHsp20* gene family in sunflower. This circular heatmap displays the relative transcript abundance of *HaHsp20* genes in various tissues across four developmental stages: seedling stage (root, leaf); juvenile stage (primary root, apical stem, basal stem, new leaf); bud stage (old leaf, lateral root); and flower stage (old leaf, apical stem, disk flower, primary root, sepal, anther). The color gradient represents normalized expression levels, with white indicating low transcript abundance and dark blue indicating high transcript abundance. The corresponding scale bar is provided at the top right.

To explore the expression pattern of the *HaHsp20* gene family in different tissues and developmental stages of sunflower, transcriptome data were used to generate expression heatmaps of the *HaHsp20* gene family across various tissues at four developmental stages (seedling, juvenile, bud, and flower stages) (Figure 5). Overall, *HaHsp20* family genes exhibited a constitutive expression pattern, and the expression levels of different *HaHsp20* genes varied considerably among stages and tissues. Most *HaHsp20* genes showed the highest expression levels in old leaves at the bud stage, such as *HaHsp17.68*, *HaHsp21.31A*, and *HaHsp17.55*. At the flower stage, the expression levels of some *HaHsp20* genes (e.g., *HaHsp29.94*, *HaHsp24.61*, and *HaHsp25.91*) were significantly elevated in disc florets and old leaves. These results provide strong evidence that the spatiotemporal expression diversification of *HaHsp20* genes underlies the complex developmental programs and tissue-specific physiological processes in sunflower.

**Figure 6:**
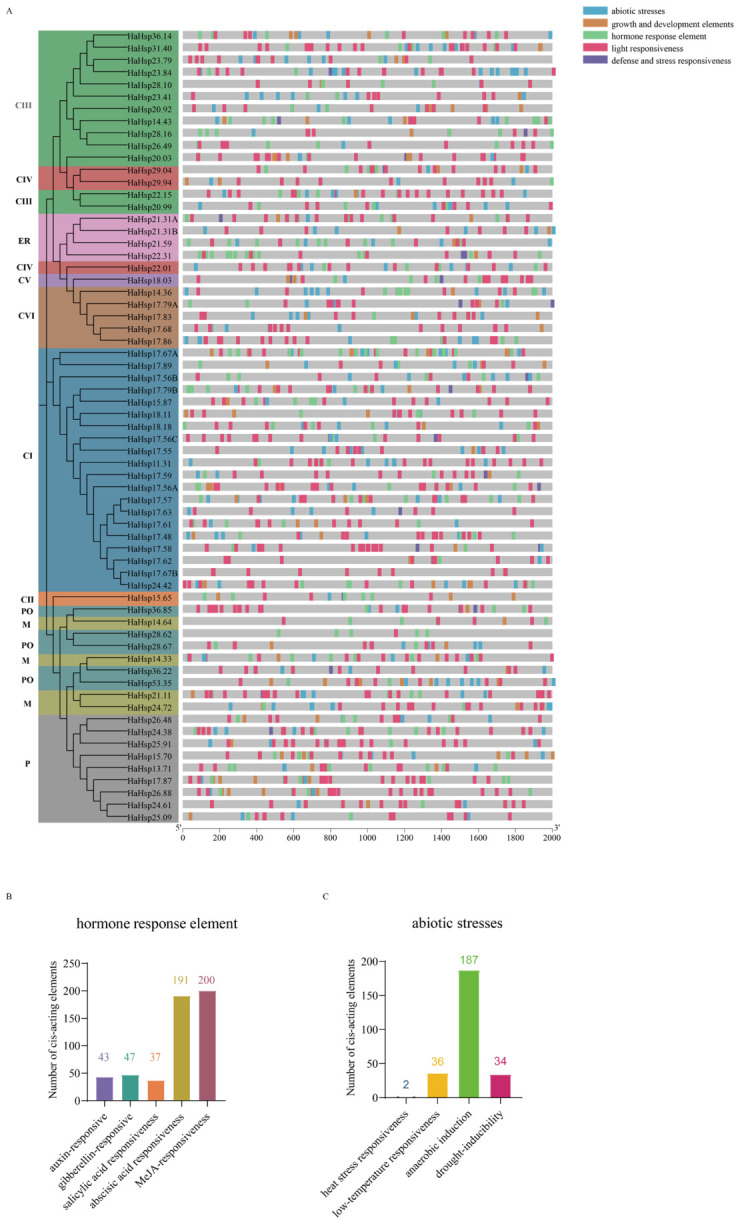
Analysis of cis-acting elements in *HaHsp20* promoters. The 2 kb upstream sequences of the *HaHsp20* transcription start site (TSS) were analyzed for cis-acting elements. (**A**) Classification of cis-acting elements in the promoter regions of *HaHsp20* genes. According to their annotations, the elements were divided into five major categories: abiotic stresses, defense and stress responsiveness, growth and development, light responsiveness, and hormone response. Different colors represent different categories. (**B**) Distribution and number of hormone-responsive cis-acting elements in the promoter regions of the 65 *HaHsp20* genes. (**C**) Distribution and number of abiotic stress-related cis-acting elements in the promoter regions of the 65 *HaHsp20* genes.

To explore the potential functions of the *HaHsp20* family genes, the cis-regulatory elements in the promoter regions (2 kb upstream of the transcription start site, TSS) of these 65 genes were analyzed (Figure 6A). Based on annotation, all identified cis-elements were classified into five categories: abiotic stresses, defense and stress responsiveness, growth and development, light responsiveness and hormone response. Analysis of the hormone response element and abiotic stress-related cis-elements in the *HaHsp20* promoters revealed a total of 518 phytohormone-related cis-elements, including 43 auxin-responsive elements, 47 gibberellin (GA)-responsive elements, 37 salicylic acid (SA) responsive elements, 191 abscisic acid (ABA)-responsive elements, and 200 MeJA-responsive elements (Figure 6B). In addition, 259 abiotic stress-related cis-elements were identified, comprising two heat stress-responsive elements, 36 low-temperature-responsive elements, 187 anaerobic induction-responsive elements, and 34 drought-responsive elements (Figure 6C). These results indicate that *HaHsp20* family genes are involved in sunflower growth and development and suggest their potential involvement in phytohormone and abiotic stress responses.

### 2.6. Expression Pattern of HaHsp20 Under Heat Stress

**Figure 7:**
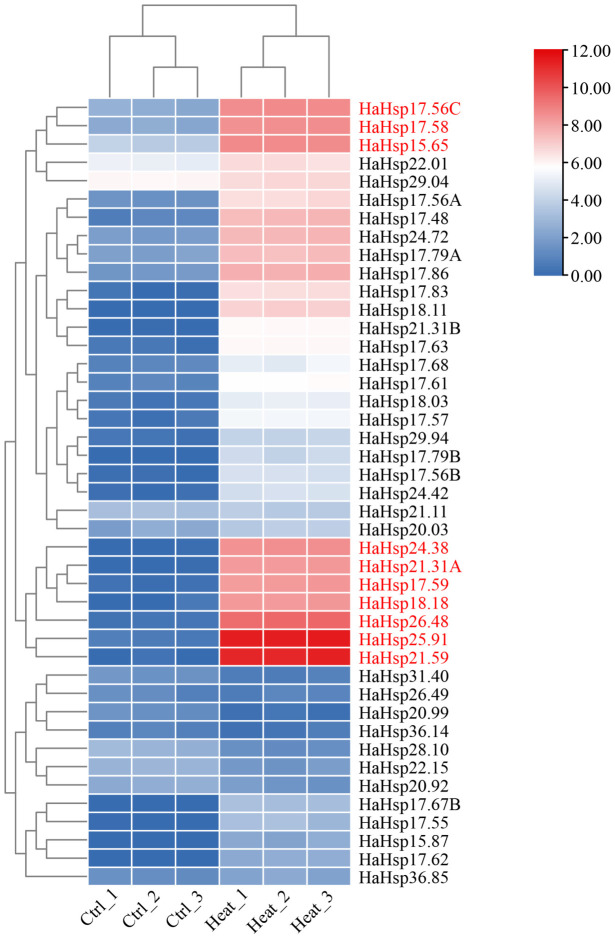
Expression profiling of the *HaHsp20* family genes under high temperature. Sunflower leaves were sampled before and after 10 h of high-temperature treatment, and RNA-seq was performed with three biological replicates. A total of 43 differentially expressed *HaHsp20* genes were identified. The heatmap was generated using TBtools based on log_2_(FPKM + 1) values with hierarchical clustering applied to both rows (genes) and columns (samples). The color gradient represents log_2_(FPKM + 1) expression levels, with red indicating high transcript abundance and blue indicating low transcript abundance. Genes highlighted in red represent *HaHsp* genes whose expression levels were significantly increased after high-temperature treatment.

To preliminarily investigate the function of *HaHsp20* genes under heat stress, six RNA-seq libraries were constructed and sequenced, including three independent biological replicates under normal temperature and under heat stress conditions at 45 °C for 10 h. A heatmap of 43 differentially expressed *HaHsp20* genes was generated using FPKM values from the RNA-seq data to estimate the expression levels of these genes (Figure 7). The heatmap showed that most *HaHsp20* genes were upregulated after 10 h of heat stress, among which *HaHsp25.91* and *HaHsp21.59* exhibited the most pronounced upregulation.

**Figure 8:**
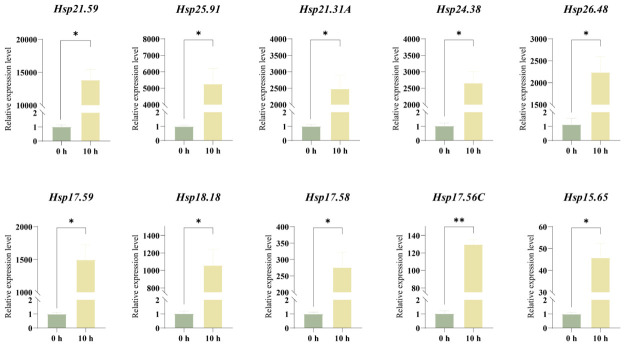
Expression patterns of *HaHsp20* genes after high-temperature treatment. Based on RNA-seq data, the differentially expressed genes showing significant changes between 0 h and 10 h of high-temperature treatment were validated by RT-qPCR. *n* = 3, for each biological replicate, three technical replicates were performed. Data are presented as the mean ± S.E.M., relative expression levels calculated using the 2^−ΔΔCt^ method, with 0 h samples serving as the calibrator (set to 1). Statistical significance was determined by a two-tailed Student’s *t*-test. * indicates a statistically significant difference at *p* < 0.05, ** indicates a statistically significant difference at *p* < 0.01. *HaTubulin* was used as the reference gene for normalization.

To further investigate the induction of *HaHsp20* genes in response to heat stress, ten significantly differentially expressed *HaHsp20* genes were selected, and RT-qPCR was performed to test their expression levels. Consistent with the RNA-seq data, all ten selected *HaHsp20* genes were significantly upregulated under heat stress (Figure 7 and Figure 8). Specifically, after 10 h of heat stress, seven of the *HaHsp20* genes (*HaHsp21.59*, *HaHsp25.91*, *HaHsp21.31A*, *HaHsp24.38*, *HaHsp26.48*, *HaHsp17.59*, and *HaHsp18.18*) were extremely upregulated (more than 1000-fold) (Figure 8). Collectively, these results demonstrate that *HaHsp20* genes are strongly and rapidly induced by heat stress, suggesting their critical roles in the heat stress response.

## 3. Discussion

In this study, we identified 65 *HaHsp20* genes in sunflower, a number substantially higher than the approximately 19 genes reported in *Arabidopsis thaliana* and 23 in rice [26,37]. The large family size in sunflower is consistent with the observation across other plants that sHsps gene numbers vary considerably among species, reflecting lineage-specific expansion events [25]. A major driver of such expansion is whole-genome duplication (WGD) and polyploidization [40]. Sunflower has experienced at least two rounds of polyploidization, including a whole-genome triplication at the base of the Asterids II clade and a sunflower-specific WGD approximately 29 million years ago [34]. These ancient genomic events likely provided the raw genetic material for the expansion and neofunctionalization of the *Hsp20* family in sunflower. A similar phenomenon has been documented in hexaploid wheat, where sHsps massively expanded in the A and B subgenomes through intrachromosomal duplications during polyploidization [41]. Collectively, these findings strongly suggest that the expansion of *HaHsp20* family members in sunflower was driven by multiple rounds of polyploidization and subsequent gene duplication events.

Phylogenetic analysis classified the HaHsp20 proteins into 10 distinct subfamilies, including CI-CVI, ER, P, PO and M. This classification largely aligns with the widely accepted 10–12 subfamily framework established for angiosperms [25]. The CI subfamily, which contains the largest number of members (*n* = 20), corresponds to the cytosolic class I sHsps that are primarily localized in the cytoplasm. This enrichment of CI members is consistent with findings in other plant species such as *Lycium barbarum* and *Paeonia suffruticosa*, where cytosolic sHsps constitute the predominant subfamily [22,42]. The substantial expansion of the CI subfamily in sunflower may reflect the crucial role of cytoplasmic sHsps in maintaining protein homeostasis under various environmental stresses [25]. Subcellular localization predictions further support this functional distribution, as the majority of HaHsp20 proteins were predicted to localize to the cytoplasm, with additional members targeted to chloroplasts, mitochondria and peroxisomes. This compartmentalization pattern underscores the specialized functions of sHsps in protecting distinct organelles from stress-induced damage [25,26].

Promoter cis-regulatory element analysis revealed a striking abundance of ABA responsive elements (*n* = 191) and MeJA-responsive elements (*n* = 200) in *HaHsp20* promoters, while heat shock elements (HSEs) were surprisingly scarce (only two identified). Abscisic acid is a key phytohormone that mediates plant responses to multiple abiotic stresses, including heat stress, by activating downstream heat shock factors and Hsp expression [43]. Similarly, MeJA has been shown to enhance thermotolerance in plants by promoting the expression of stress-responsive genes [44]. In *Agrostis stolonifera*, the sHsp AsHSP26.8a was found to modulate plant abiotic stress responses through ABA-dependent and ABA-independent signaling pathways [45]. Likewise, in barley, in silico cis-regulatory motif analysis of HSF promoters revealed enrichment of ABRE elements, indicating a regulatory role of ABA in mediating the transcriptional response of *HSF* genes [46]. These observations collectively indicate that ABA and MeJA play pivotal roles in regulating the heat-responsive expression of *HaHsp20* genes in sunflower. The presence of numerous drought- and low-temperature-responsive cis-elements further suggests that *HaHsp20* genes may be involved in multiple abiotic stress responses, consistent with the known functional diversity of sHsps [47]. However, whether these elements are functionally active and whether they indeed mediate heat responsive expression remain to be experimentally tested.

The expression pattern analysis revealed that most *HaHsp20* genes exhibited the highest transcript abundance in old leaves at the bud stage, whereas expression levels in other tissues varied. This pattern is consistent with findings in other plant species, where sHsps have been implicated in leaf senescence processes. In *Arabidopsis*, several sHsps genes are upregulated during natural leaf senescence and Hsp17.6B has been functionally annotated to be involved in the response to oxidative stress, which is a hallmark of senescing leaves [48,49]. In rice, the aging-responsive heat shock protein OsHSP18.2 was identified as an aging-responsive protein, and its functional analysis demonstrated that sHsps improve seed vigor and longevity by restricting ROS accumulation during the aging process [50]. The elevated expression of *HaHsp20* genes in old leaves may serve as a protective mechanism to counter the accumulation of reactive oxygen species and protein damage during leaf senescence, thereby maintaining cellular homeostasis in aging tissues [24].

Under heat stress conditions, the vast majority of differentially expressed *HaHsp20* genes were significantly upregulated, with *HaHsp21.59* and *HaHsp25.91* exhibiting the most pronounced induction (more than 4000-fold). This dramatic upregulation strongly indicates that these two genes are core heat-responsive factors in sunflower. The robust expression of sHsps under heat stress is a well-conserved feature across the plant kingdom, as sHsps function as ATP-independent molecular chaperones that bind denatured or misfolded substrate proteins, preventing irreversible aggregation and maintaining protein homeostasis [23,24]. Overexpression of sHsps has been shown to confer enhanced thermotolerance in multiple plant species. For instance, transgenic *Arabidopsis* plants overexpressing *CsHSP17.7*, *CsHSP18.1* and *CsHSP21.8* from *Camellia sinensis* exhibited lower malondialdehyde content, ion leakage, higher proline content and upregulated expression of stress-related genes [51]. Similarly, overexpression of *PtsHSP17.2* from *Pinellia ternata* in tobacco enhanced heat tolerance, alleviated oxidative stress, and increased antioxidant enzyme activity and proline content [52]. In rice, overexpression of *sHSP17.7* conferred both heat tolerance and UV-B resistance [53]. These functional validations across diverse plant species reinforce the protective role of sHsps under heat stress and suggest that *HaHsp21.59* and *HaHsp25.91* are promising candidate genes for future functional validation of their potential roles in heat stress tolerance.

## 4. Materials and Methods

### 4.1. Identification of HaHsp20 Gene Family Members in Sunflower

The genomic data (including protein coding sequence files, gene sequence files, and gene annotation files) of sunflower and rice were downloaded from the NCBI database (National Center for Biotechnology Information, https://www.ncbi.nlm.nih.gov, accessed on 15 May 2026). *Arabidopsis thaliana* genomic data were obtained from the TAIR website (TAIR; https://www.arabidopsis.org/, accessed on 15 May 2026). To identify all potential candidate *HaHsp20* family members in sunflower, the hidden Markov model (HMM) profile of the α-crystallin domain (ACD, PF00011) was downloaded from the Pfam database, and a search against the sunflower proteome was performed using hmm search (HMMER 3.3.2) with an E-value threshold of ≤1 × 10^−5^. To avoid missing any HaHsp20 sequences, BLASTp searches (E-value ≤ 1 × 10^−5^) were also conducted using the *Arabidopsis* Hsp20 protein sequences obtained from TAIR as queries. The sequences obtained from both methods were combined and redundant sequences were removed to obtain candidate protein sequences. Finally, the candidate protein sequences were submitted to the NCBI Conserved Domain Database (CDD), Pfam, and SMART to verify the presence of the ACD; sequences lacking a typical ACD were discarded. The remaining non-redundant, high-confidence genes were defined as members of the *HaHsp20* gene family and were named according to their molecular weights. For gene loci with multiple transcript variants, the longest transcript was retained as the representative sequence for subsequent analyses, and its transcript ID is reported in Table 1. Detailed information on HaHsp20 is provided in Table S1.

### 4.2. Analysis of Physicochemical Properties and Subcellular Localization

Physicochemical properties, including amino acid number, molecular weight (MW), theoretical isoelectric point (pI), and instability index, of HaHsp20 proteins were analyzed using the Protein Parameter Calc plugin in TBtools. v2.485 Subcellular localization of HaHsp20 proteins was predicted using the WoLF PSORT online software (https://wolfpsort.hgc.jp/, accessed on 19 May 2026).

### 4.3. Chromosomal Localization, Conserved Domain, and Phylogenetic Analysis

Chromosomal location information of the *HaHsp20* gene family members was extracted from the genomic GFF file and visualized using TBtools v2.485. Functional domains of the members were searched using the NCBI Conserved Domain Database (CDD, https://www.ncbi.nlm.nih.gov/cdd/, accessed on 18 May 2026). For phylogenetic analysis, full-length Hsp20 amino acid sequences of *Arabidopsis* [26] and rice [37] were downloaded from public databases. Multiple sequence alignments of HaHsp20 protein sequences from sunflower, rice, and *Arabidopsis* were performed using MEGA (version 11.0). A phylogenetic tree was constructed using the Maximum Likelihood (ML) method with 1000 bootstrap replicates, and all other parameters were set to default. The resulting phylogenetic tree was visualized, beautified, and annotated using the iTOL online website (https://itol.embl.de, accessed on 20 May 2026). Subgroup classification was performed based on the topology of the phylogenetic tree and the Hsp20 classification methods of the reference species [26].

### 4.4. Gene Structure and Conserved Motif Analysis

Exon and intron data of *HaHsp20* genes were obtained from NCBI. Conserved motifs in HaHsp20 protein sequences were identified using MEME (https://meme-suite.org/meme/tools/meme, accessed on 18 May 2026) with the following parameters: any number of repetitions, a maximum of 10 motifs, and an optimal motif width of 6–50 amino acid residues. Finally, visualization analysis was performed using TBtools v2.485.

### 4.5. Analysis of Synteny Analysis, Ka/Ks and Promoter Cis-Regulatory Element

To further analyze the evolutionary relationships among *HaHsp20* family members within the sunflower genome, intraspecies synteny analysis was performed using the Advanced Circos and Dual Synteny Plot plugins in TBtools v2.485.

To evaluate the selective pressures acting on duplicated *HaHsp* gene pairs, non-synonymous (Ka) and synonymous (Ks) substitution rates were calculated using the Simple Ka/Ks Calculator implemented in TBtools v2.485. Coding sequences (CDSs) of each duplicated gene pair were aligned using the built-in alignment function of TBtools v2.485. Ka/Ks ratios <1, =1, and >1 were interpreted as purifying selection, neutral evolution, and positive selection, respectively. The detailed results are provided in Table 2.

The 2000 bp sequence upstream of the transcription start site (TSS) for each *HaHsp20* gene was extracted from the sunflower genome file using TBtools v2.485 and submitted to the PlantCARE database (https://bioinformatics.psb.ugent.be/webtools/plantcare/html/, accessed on 19 May 2026) for cis-element prediction [54]. The predicted cis-elements were systematically classified according to their functional annotations. Results were visualized using TBtools v2.485.

### 4.6. Plant Materials and Heat Treatments

To investigate the expression patterns of *HaHsp20* genes under heat stress, sunflower seeds were grown in soil (soil:vermiculite = 3:1) for one month under controlled conditions (25 °C, 16 h light/8 h dark photoperiod). The growth chamber temperature was then increased to 45 °C to impose heat stress. Leaf samples from three sunflower plants were collected at 0 h and 10 h of heat treatment (three biological replicates per time point), immediately frozen on dry ice, and stored at −80 °C until RNA extraction. Total RNA was extracted using the RNA-easy Isolation Reagent (Vazyme Biotech Co., Ltd., Nanjing, China, catalog No. R701) according to the manufacturer’s instructions. RNA quality and integrity were assessed using a NanoDrop 2000 spectrophotometer (Thermo Fisher Scientific, Waltham, MA, USA) and an Agilent 2100 Bioanalyzer (Agilent Technologies, Santa Clara, CA, USA). cDNA libraries were constructed following the Illumina protocol: mRNA was enriched using Oligo(dT)-coated magnetic beads, fragmented, and reverse-transcribed into cDNA. The cDNA was then end-repaired, A-tailed, ligated to sequencing adapters, size-selected using AMPure XP beads, and PCR-amplified to generate the final libraries. Library quality and concentration were validated using a Qubit 3.0 fluorometer (Thermo Fisher Scientific, Waltham, MA, USA), a Qsep400 high-throughput analysis system, and Q-PCR. Sequencing was performed on an Illumina platform (Biomarker Technologies, Beijing, China) with a paired-end 150 bp (PE150) read configuration.

Raw reads were processed using the BMKCloud platform (www.biocloud.net) with the following filtering criteria: (1) removal of adapter sequences; (2) removal of reads with more than 40% of bases having a quality score (Q) < 15; (3) removal of reads with polyG tails; (4) removal of reads with complexity < 10%; (5) removal of reads shorter than 100 bp; and (6) removal of reads with more than 5 ambiguous bases (N) in a single-end read. After filtering, a total of 144.82 Gb of clean data were obtained, with each sample yielding at least 6.02 Gb of clean data and Q30 values ≥ 94.99%. Clean reads were aligned to the sunflower reference genome (GCA_002127325.2) using HISAT2 v2.1.0 [55]. Aligned reads were assembled and quantified using StringTie v1.3.3 [56], and gene expression levels were calculated as FPKM (Fragments Per Kilobase of transcript per Million mapped fragments). Differentially expressed genes (DEGs) between 0 h and 10 h heat-treated samples were identified using DESeq2 v1.30.0 with a fold-change threshold of |log_2_FC| ≥ 2 and an FDR < 0.01. The mapping efficiency for each sample ranged from 90.84% to 93.80%. Transcriptome data are listed in Table S2.

### 4.7. Analysis of Expression Patterns of the HaHsp20 Gene Family in Different Tissues

To investigate the expression levels of the *HaHsp20* genes, the developmental expression pattern of *HaHsp20* genes was evaluated at key growth stages of sunflower. Sampling covered various tissues from four developmental stages, including the seedling stage (root, leaf), juvenile stage (primary root, apical stem, basal stem, new leaf), bud stage (old leaf, lateral root), and flowering stage (old leaf, apical stem, disk flower, primary root, sepal, anther). Detailed data are presented in Table S3. The Transcriptome sequencing was performed by Biomarker Technologies to obtain gene expression levels, and a heatmap reflecting the gene expression patterns was generated using TBtools v2.485 software based on FPKM values.

### 4.8. RNA Extraction and RT-qPCR Analysis

Total RNA was extracted from leaves of sunflower plants treated at 45 °C for 0 h and 10 h using the RNA-easy Isolation Reagent (Vazyme Biotech Co., Ltd., Nanjing, China, catalog No. R701) according to the manufacturer’s instructions. Subsequently, the isolated RNA was reverse-transcribed into cDNA using the HiScript II Q RT SuperMix (Vazyme Biotech Co., Ltd., Nanjing, China, catalog No. R223) for RT-qPCR analysis. RT-qPCR was performed using the CFX384 Touch Real-Time PCR Detection System (Bio-Rad Laboratories, Hercules, CA, USA) and ChamQ Universal SYBR qPCR Master Mix (Vazyme Biotech Co., Ltd., Nanjing, China, catalog No. Q711) in a 20 µL reaction volume containing 10 µL of 2× SYBR Green Master Mix, 0.4 µM of each primer, and 2 µL of diluted cDNA (1:10). The thermal cycling protocol was as follows: 95 °C for 30 s, followed by 40 cycles of 95 °C for 5 s and 60 °C for 30 s. Primer specificity was ensured by BLAST analysis (NCBI BLAST web server (https://blast.ncbi.nlm.nih.gov, accessed on 20 May 2026) against the sunflower genome database and by designing all primer pairs with similar annealing temperatures. *HaTubulin* was used as the reference gene, and its expression level remained stable across all samples. The primers used are listed in Supplementary Table S1. Relative gene expression levels were calculated using the 2^−ΔΔCt^ method, with the 0 h samples serving as the calibrator. Each experiment was performed with three biological replicates and three technical replicates. Statistical significance was determined using a two-tailed Student’s *t*-test comparing each treated sample (10 h) to the corresponding control (0 h), with a significance threshold of *p* < 0.05. All statistical analyses were performed using GraphPad Prism 8.0. The primer sequences used in this study are listed in Table S4.

## 5. Conclusions

In this study, a total of 65 *HaHsp20* genes were systematically identified in sunflower. The expansion of this family was primarily driven by segmental duplication events, with the cytoplasmic CI subfamily representing the most amplified clade. Most *HaHsp20* genes exhibited high expression levels in old leaves and were significantly upregulated under heat stress, particularly *HaHsp21.59* and *HaHsp25.91* (more than 4000-fold). Promoter analysis revealed a large number of ABA and MeJA-responsive elements but few canonical heat shock elements, suggesting a possible involvement of phytohormone signaling in the heat-inducible expression of these genes, a hypothesis that requires experimental validation. These findings provide a comprehensive foundation for understanding the evolutionary history and expression dynamics of the *HaHsp20* family in sunflower, and highlight *HaHsp21.59* and *HaHsp25.91* as promising candidate genes for future functional validation of their potential roles in heat stress tolerance.

## Figures and Tables

**Table 1:** Basic physicochemical properties of Hsp20 proteins.

Name	Transcript ID	Number of Amino Acid	Molecular Weight	pI	Instability Index	Aliphatic Index	Grand Average of Hydropathicity	Subcellular Localization
HaHsp36.14	rna-XM_022137079.2	326	36,145.44	9.75	44.64	67.21	−0.863	Chloroplast
HaHsp20.99	rna-XM_022181885.2	185	20,995.27	9.7	42.67	93.68	−0.296	Cytoplasmic
HaHsp14.36	rna-XM_022174995.1	125	14,367.56	9.67	61.02	73.12	−0.729	Cytoplasmic
HaHsp31.40	rna-XM_035974847.1	280	31,400.38	9.59	70.32	59.82	−0.943	Chloroplast
HaHsp28.67	rna-XM_035985974.1	247	28,673.37	9.57	35.12	80.04	−0.41	Peroxisome
HaHsp28.62	rna-XM_035984823.1	247	28,623.33	9.54	37.65	80.04	−0.4	Peroxisome
HaHsp26.48	rna-XM_022120244.2	230	26,481.29	9.54	46.66	66.91	−0.896	Chloroplast
HaHsp53.35	rna-XM_035984822.1	462	53,355.94	9.48	44.41	80.97	−0.301	Chloroplast
HaHsp14.33	rna-XM_022120043.2	129	14,331.47	9.4	60.51	97.44	−0.299	Mitochondrion
HaHsp23.79	rna-XM_022177505.2	211	23,795.63	9.18	31.76	89.05	−0.309	Cytoplasmic
HaHsp23.84	rna-XM_022178367.2	211	23,841.78	9.17	34.81	95.55	−0.239	Cytoplasmic
HaHsp24.42	rna-XM_022117494.2	214	24,426.07	9.13	54.73	82.34	−0.442	Cytoplasmic
HaHsp36.22	rna-XM_022120081.2	318	36,220.03	8.96	54.51	72.36	−0.398	Chloroplast
HaHsp24.38	rna-XM_022142383.2	214	24,386.9	8.85	40.92	69.72	−0.725	Chloroplast
HaHsp29.04	rna-XM_022130561.2	262	29,047.08	8.79	61.83	75.57	−0.398	Nucleus
HaHsp18.18	rna-XM_022114744.2	158	18,186.81	8.64	45.34	77.09	−0.602	Cytoplasmic
HaHsp24.61	rna-XM_022177467.2	214	24,610.84	8.55	60.08	71.96	−0.835	Mitochondrion
HaHsp26.88	rna-XM_022176126.2	234	26,888.43	8.53	50	76.62	−0.782	Chloroplast
HaHsp17.79A	rna-XM_022172551.2	160	17,796.46	8.49	38.64	77.94	−0.532	Cytoplasmic
HaHsp21.59	rna-XM_022113785.2	191	21,593.86	7.92	25.78	88.27	−0.428	Chloroplast
HaHsp22.15	rna-XM_022138766.2	196	22,159.92	7.76	50.25	67.04	−0.726	Golgi apparatus
HaHsp28.10	rna-XM_022155395.2	253	28,100.83	7.71	32.36	75.53	−0.691	Cytoplasmic
HaHsp23.41	rna-XM_022161941.2	207	23,415.65	7.63	42.36	73.86	−0.694	Cytoplasmic
HaHsp29.94	rna-XM_022165941.2	266	29,942.91	7.08	61.73	74.02	−0.532	Chloroplast
HaHsp25.09	rna-XM_022177468.2	219	25,098.4	7.07	58.62	74.34	−0.8	Chloroplast
HaHsp25.91	rna-XM_022121879.2	232	25,910.37	6.91	54.57	72.67	−0.623	Chloroplast
HaHsp17.56A	rna-XM_022178616.2	155	17,562.88	6.77	58.99	74.06	−0.657	Cytoplasmic
HaHsp17.68	rna-XM_022172554.2	159	17,683.14	6.62	36.88	74.78	−0.589	Cytoplasmic
HaHsp36.85	rna-XM_022133189.2	330	36,858.8	6.54	43.36	87.94	−0.134	Chloroplast
HaHsp21.31A	rna-XM_022131458.2	184	21,314.7	6.45	42.44	87.45	−0.484	Chloroplast
HaHsp14.43	rna-XM_022123285.2	125	14,438.41	6.42	54.65	80.96	−0.726	Cytoplasmic
HaHsp15.65	rna-XM_022143263.2	140	15,656.95	6.31	38.64	88.29	−0.399	Peroxisome
HaHsp22.01	rna-XM_022155446.2	192	22,010.14	6.23	49.66	86.25	−0.124	Cytoplasmic
HaHsp17.67A	rna-XM_022150703.2	156	17,673.06	6.19	50.28	74.94	−0.613	Cytoplasmic
HaHsp17.48	rna-XM_022117530.2	155	17,487.81	6.19	55.36	74.06	−0.635	Cytoplasmic
HaHsp17.57	rna-XM_022117559.2	155	17,577.89	6.19	57.56	74.71	−0.655	Cytoplasmic
HaHsp17.86	rna-XM_022172556.2	160	17,865.36	6.16	33.1	79.12	−0.537	Cytoplasmic
HaHsp21.31B	rna-XM_022140968.2	187	21,312.55	6.12	48.51	86.1	−0.461	Chloroplast
HaHsp18.11	rna-XM_022114741.2	158	18,105.61	6.12	40.54	77.66	−0.595	Cytoplasmic
HaHsp17.67B	rna-XM_022170330.2	155	17,670.98	5.98	57.55	74.71	−0.677	Cytoplasmic
HaHsp18.03	rna-XM_022141354.2	163	18,038.64	5.97	54.66	93.74	−0.375	Nucleus
HaHsp20.92	rna-XM_022137515.2	180	20,920	5.97	48.02	77.33	−0.431	Cytoplasmic
HaHsp26.49	rna-XM_022161830.2	231	26,490.33	5.94	49.75	63.33	−1.097	Nucleus
HaHsp17.55	rna-XM_022117244.2	155	17,555.93	5.82	57.99	77.87	−0.615	Cytoplasmic
HaHsp17.56C	rna-XM_035977026.1	155	17,560.86	5.82	56.06	75.35	−0.645	Cytoplasmic
HaHsp17.63	rna-XM_022117589.2	155	17,635.93	5.82	51.64	72.19	−0.668	Cytoplasmic
HaHsp15.87	rna-XM_022143023.2	138	15,873.21	5.82	48.15	65.58	−0.688	Cytoplasmic
HaHsp17.58	rna-XM_022117584.2	155	17,584.93	5.81	52.18	76	−0.652	Cytoplasmic
HaHsp17.61	rna-XM_022166447.2	155	17,619.95	5.81	56.33	72.84	−0.677	Cytoplasmic
HaHsp17.83	rna-XM_022172550.2	160	17,830.36	5.76	35.56	84.62	−0.495	Cytoplasmic
HaHsp17.87	rna-XM_022176123.2	158	17,873.41	5.72	37.84	73.42	−0.715	Cytoplasmic
HaHsp17.89	rna-XM_022150704.2	157	17,895.15	5.59	61.96	69.49	−0.732	Cytoplasmic
HaHsp17.79B	rna-XM_022143022.2	155	17,794.3	5.57	48.61	76	−0.6	Cytoplasmic
HaHsp17.59	rna-XM_022117571.2	155	17,592.96	5.55	50.76	74.06	−0.585	Cytoplasmic
HaHsp17.62	rna-XM_022117596.2	155	17,623.85	5.55	55.95	70.32	−0.706	Cytoplasmic
HaHsp28.16	rna-XM_022161453.2	248	28,169.13	5.54	50.31	65.32	−1.015	Chloroplast
HaHsp14.64	rna-XM_035987155.1	128	14,641.85	5.51	41.16	82.89	−0.441	Cytoplasmic
HaHsp24.72	rna-XM_022173992.2	218	24,724.8	5.46	60.79	72.02	−0.682	Chloroplast
HaHsp22.31	rna-XM_022168179.2	197	22,319.43	5.36	33.65	86.04	−0.477	Chloroplast
HaHsp17.56B	rna-XM_022118150.2	153	17,562.04	5.25	51.99	73.86	−0.629	Cytoplasmic
HaHsp11.31	rna-XM_022163361.1	100	11,312.64	5.07	53.44	70.1	−0.833	Cytoplasmic
HaHsp21.11	rna-XM_022146609.2	185	21,118.83	5.05	49.49	82.16	−0.558	Chloroplast
HaHsp13.71	rna-XM_035989716.1	118	13,712.57	5.02	37.75	73.47	−0.803	Mitochondrion
HaHsp15.70	rna-XM_022177466.2	137	15,701.81	4.95	32.05	82.48	−0.591	Cytoplasmic
HaHsp20.03	rna-XM_022115349.2	190	20,038.61	4.71	21.88	84.63	−0.068	Cytoplasmic

**Table 2:** Ka, Ks, and Ka/Ks values for the duplication gene pairs from sunflower.

Seq_1	Seq_2	Ka	Ks	Ka/Ks	Duplication Type
*HaHsp15.87*	*HaHsp18.11*	0.161	1.334	0.121	WGD or segmental duplication
*HaHsp20.99*	*HaHsp22.15*	0.209	0.608	0.344	WGD or segmental duplication
*HaHsp24.72*	*HaHsp21.11*	0.251	0.678	0.370	WGD or segmental duplication
*HaHsp17.56B*	*HaHsp17.89*	0.114	0.921	0.124	WGD or segmental duplication
*HaHsp26.48*	*HaHsp24.38*	0.143	0.367	0.390	WGD or segmental duplication
*HaHsp14.43*	*HaHsp28.16*	0.148	0.564	0.262	WGD or segmental duplication
*HaHsp28.10*	*HaHsp23.41*	0.339	1.337	0.254	WGD or segmental duplication
*HaHsp29.04*	*HaHsp29.94*	0.180	0.447	0.403	WGD or segmental duplication

## Data Availability

The original contributions presented in this study are included in the article/Supplementary Materials. Further inquiries can be directed to the corresponding authors.

## Supplementary Materials

The following supporting information can be downloaded at: https://www.mdpi.com/article/10.3390/ijms27135799/s1.
